# Rheumatoid meningitis: a rare neurological complication of rheumatoid arthritis

**DOI:** 10.3389/fimmu.2023.1065650

**Published:** 2023-06-07

**Authors:** Siyuan Fan, Jiuliang Zhao, Bo Hou, Mange Liu, Jingwen Niu, Yan Zhou, Chenhui Mao, Haitao Ren, Feng Feng, Mengtao Li, Xiaofeng Zeng, Yicheng Zhu, Hongzhi Guan

**Affiliations:** ^1^ Department of Neurology, Peking Union Medical College Hospital, Chinese Academy of Medical Sciences and Peking Union Medical College, Beijing, China; ^2^ Department of Rheumatology and Clinical Immunology, Chinese Academy of Medical Sciences and Peking Union Medical College, Beijing, China; ^3^ National Clinical Research Center for Dermatologic and Immunologic Diseases, Ministry of Science and Technology, Beijing, China; ^4^ State Key Laboratory of Complex Severe and Rare Diseases, Peking Union Medical College Hospital, Beijing, China; ^5^ Key Laboratory of Rheumatology and Clinical Immunology, Ministry of Education, Beijing, China; ^6^ Department of Radiology, Peking Union Medical College Hospital, Chinese Academy of Medical Sciences and Peking Union Medical College, Beijing, China

**Keywords:** rheumatoid arthritis, rheumatoid meningitis, immunotherapy, tocilizumab (TCZ), neuroimage

## Abstract

**Objective:**

To describe the clinical and neuroimaging characteristics of rheumatoid meningitis (RM) in Chinese patients.

**Methods:**

The patients admitted to our hospital with the diagnosis of RM in the past 8 years were retrospectively analyzed.

**Results:**

Six patients with RM were identified among 933 patients admitted with rheumatoid arthritis (RA). The symptoms of meningitis occurred after onset of arthritis in five patients and before onset in one. Headache (n=6), hyperacute focal neurological deficits (n=4) and seizures (n=3) were the most prevalent symptoms. The nadir modified Rankin Scale score was ≥3 in five patients. Rheumatoid factor was elevated in all patients, and interleukin-6 levels in cerebrospinal fluid were dramatically elevated in three of four tested patients. Magnetic resonance imaging of the brain revealed that the meninges were affected in all patients and the cerebral parenchyma was affected in one patient. The lesions were generally located in the frontoparietal region and showed restricted diffusion along the adjacent subarachnoid space. RM occurred during disease-modifying therapy in four patients. In the acute episode, three patients improved on tocilizumab and the other three improved on pulse corticosteroids. For maintenance therapy, two patients received combined therapy of tocilizumab and other immunosuppressive agents, one received adalimumab and methotrexate, and two received low-dose oral corticosteroids with an immunosuppressive agent. Five patients had a good outcome, and one died of *Pneumocystis jirovecii* pneumonia after stabilization of his neurologic conditions. No relapse of RM occurred on immunotherapy during follow-up.

**Conclusions:**

Chinese patients with RM share some remarkable clinical and neuroimaging features and respond well to appropriate immunotherapy. Tocilizumab could be a treatment option for this severe complication of RA.

## 1 Introduction

Rheumatoid meningitis (RM) is a rare but severe neurological complication of rheumatoid arthritis (RA) and has a predilection for the meninges rather than the brain parenchyma (1). Neurological manifestations of RM include headache, cranial nerve palsy, seizure, altered mental status, and focal neurologic deficits. Some patients with RM present with acute focal neurologic deficits, which may be initially misdiagnosed as acute ischemic stroke (2–8). Some patients with RA develop these symptoms when still on disease-modifying therapies (DMTs), such as biological agents and immunosuppressive agents, thus necessitating differential diagnosis of central nervous system (CNS) opportunistic infections and drug-induced meningitis (7). RM can occur either before or many years after onset of arthritis (9), and the severity of CNS involvement correlates poorly with severity of systemic arthritis (2, 10), which renders the diagnosis of RM even more challenging. Meningeal biopsy may support a diagnosis of RM. However, it is invasive and sometimes has nonspecific findings (11). Some specific neuroimaging manifestations have been described but require further investigation.

RM is rarely reported in Chinese patients (12, 13). In this study, we retrospectively investigated the clinical, laboratory, and neuroimaging features and the treatment provided and outcomes in six patients with RM admitted to our hospital. Our aim was to characterize Chinese patients with RM and provide new evidence for the diagnosis and treatment of RM.

## 2 Materials and methods

This single-center retrospective case series study was conducted at the Peking Union Medical College Hospital, a tertiary referral center in Beijing, China. Patients with encephalitis or meningitis of unknown origin may be referred to our encephalitis center, known as the National Center for Autoimmune Encephalitis Quality Improvement.

We retrospectively analyzed patients admitted to our hospital with a diagnosis of “rheumatoid meningitis” between January 2013 and June 2021. The search string used for retrieving relevant medical information from the electronic health records was (“rheumatoid”) AND (“meningitis” OR “encephalitis” OR “meningoencephalitis” OR “meninges” OR “brain”). The patients were required to fulfill the 2010 American College of Rheumatology/European League Against Rheumatism classification criteria for RA. The diagnosis of RM was reviewed by a panel of specialists, including neurologists and rheumatologists, based on clinical and laboratory findings. The Disease Activity Score-28 for Rheumatoid Arthritis with ESR (DAS 28) was evaluated and calculated by rheumatologists and the modified Rankin Scale (mRS) was evaluated by neurologists. In this study, pulse corticosteroids were defined as a methylprednisolone dosage of more than 250 mg per day (or its equivalent) for several days. Tocilizumab was administered intravenously at a dose of 8 mg/kg.

All individual-level medical information, including demographic, clinical, laboratory, and neuroimaging findings, treatments, and outcome data, were retrieved from the electronic health records.

The study was approved by the institutional review board of Peking Union Medical College Hospital (S-K1747). Written informed consent for treatment with tocilizumab or pulse corticosteroids was given by each patients’ legal surrogate. Patient consent for publication was not required because de-identified data were used in the study.

### 2.1 Data availability

Data related to this study can be made available on request to the corresponding author.

## 3 Results

### 3.1 Clinical characteristics

**Figure 1:**
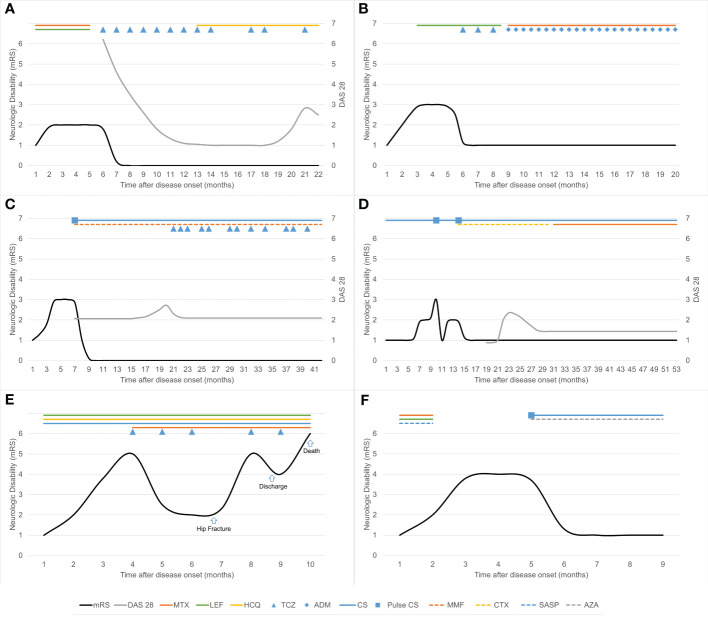
Clinical course and immunotherapy in six patients with rheumatoid meningitis. Neurologic disability was measured using the modified Rankin Scale (mRS). **(A)** In case 1, treatment with tocilizumab was transiently interrupted because of herpes zoster and surgery for gallbladder stones. **(B)** In case 2, treatment with tocilizumab was replaced by adalimumab after occurrence of skin rash. **(C)** In case 3, tocilizumab was administered once every 1–2 months because of recurrence of articular symptoms. Maintenance corticosteroid therapy was prednisone up to 10 mg daily orally. **(D)** In case 4, the maintenance corticosteroid dose was prednisone up to 10 mg daily (or its equivalent) orally. **(E)** In case 5, treatment with tocilizumab was transiently interrupted as a result of hip fracture surgery. **(F)** In case 6, the maintenance corticosteroid dose was methylprednisolone 12 mg daily orally. ADM, adalimumab; AZA, azathioprine; CS, corticosteroids; CTX, cyclophosphamide; DAS 28, Disease Activity Score-28 for Rheumatoid Arthritis with ESR; HCQ, hydroxychloroquine; LEF, leflunomide; MMF, mycophenolate mofetil; MTX, methotrexate; SASP, sulfasalazine; TCZ, tocilizumab.

Six patients (0.6%) with RM were identified among 933 patients admitted to hospital with RA during the study period. Their clinical features are summarized in **Table 1** and **Figure 1**. Five of the six patients were female. The age at onset of RM ranged from 33 to 64 years. The time interval between onset of meningeal symptoms and the last follow-up ranged from 6 to 48 months. The symptoms of meningitis or meningoencephalitis occurred after the onset of arthritis in five patients and before the onset of arthritis in one patient (case 2). Meanwhile, active arthritis was absent in one patient (case 3, the DAS 28 score was 2.06) at onset of RM.

**Table 1:** The clinical features of the six patients with rheumatoid meningitis.

	Case 1	Case 2	Case 3	Case 4	Case 5	Case 6
Sex	Female	Female	Female	Female	Male	Female
Age at disease onset, y	43	53	33	52	64	47
RA duration before RM, y	12	0	2.5	10	30	20
DMT before RM	MTX, LEF	None	None	CS	CS, LEF, HCQ	LEF, MTX, SASP
DAS 28 at disease onset	6.21	NA	2.06	NA	NA	4.98
Neurologic symptoms	Headache, dizziness	Headache, recurrent episodes of slurred speech with left-sided numbness, diplopia	Headache, recurrent episodes of slurred speech with left-sided weakness and numbness, recurrent episodes of right-sided numbness, seizures	Cognitive decline, seizures, recurrent episodes of left-sided weakness and numbness, headache	Headache, recurrent episodes of left-sided weakness and numbness, persistent weakness in the left upper and lower limbs, altered mental status	Seizures, cognitive decline, hallucinations, headache, dizziness, tinnitus, unsteady gait, urinary incontinence
Other extra-articular symptoms	Fever	None	None	None	None	None
Time from Sx to Dx, m	4	5	6	12	3	4
mRS at nadir	2	3	3	3	5	4
DMT after RM	TCZ, HCQ	TCZ, LEF, MTX, ADM	CS, MMF, TCZ	CS, CTX, MTX	CS, LEF, HCQ, TCZ, MTX	CS, AZA
Antimicrobial drugs administered	Ceftriaxone	None	Acyclovir	None	MRP, LFX, RMP	None
F/U duration, m	17	16	36	48	6	10
mRS at last F/U	0	1	0	1	6	1

ADM, adalimumab; AZA, azathioprine; CS, corticosteroids; CTX, cyclophosphamide; DMT, disease-modifying therapy; Dx, diagnosis; F/U, follow-up; HCQ, hydroxychloroquine; LEF, leflunomide; LFX, levofloxacin; m, month; MMF, mycophenolate mofetil; MRP, Meropenem; mRS, modified Rankin Scale; MTX, methotrexate; RA, rheumatoid arthritis; RM, rheumatoid meningitis; RMP, rifampicin; SASP, sulfasalazine; Sx, symptoms; TCZ, tocilizumab; y, year.

Headache occurred in all six patients and was the first neurological symptom in four. Four patients presented with hyperacute focal neurological deficits (onset within several minutes). One patient (case 5) initially presented with recurrent episodes of left-sided weakness and numbness and then progressed to persistent hemiplegia in the left upper and lower limbs. Three patients presented with epileptic seizures and one presented with cranial nerve palsy. The nadir mRS score was ≥3 in five patients.

The diagnosis of RM in these patients was delayed, with a range of 3 to 12 months from the onset of symptoms. The initial diagnosis was acute ischemic stroke in two patients (Cases 2 and 4), bacterial meningitis in one (Case 1), tuberculous meningitis in one (Case 5), viral meningitis in one (Case 3), and autoimmune encephalitis in one (Case 6).

### 3.2 Laboratory and neuroimaging findings

The laboratory findings in these patients are summarized in **Table 2**. Rheumatoid factor was elevated in all patients. Furthermore, all six patients were positive for anticitrullinated peptide antibody and five were positive for antiperinuclear factor and antikeratin antibody. The erythrocyte sedimentation rate was elevated in five patients. Blood interleukin (IL)-6 levels were normal in two of three tested patients.

**Table 2:** The laboratory findings of the six patients with rheumatoid meningitis.

	Case 1	Case 2	Case 3	Case 4	Case 5	Case 6
Peripheral blood^†^						
RF (IU/mL)	52	74	75	36.4	234	76
ACPA	+	+	+	+	+	+
APF	+	+	+	+	+	−
AKA	+	+	+	+	+	−
ESR (mm/h)	58	85	8	29	89	36
hsCRP (mg/L)	11.31	33.44	4.27	3.67	166.63	NA
IgG (g/L)	8.41	8.64	13.65	7.89	9.25	9.96
CD19+ B cell count (/μL)	180	NA	237	110	60	NA
CD4+ T cell count (/μL)	680	NA	996	341	641	NA
CD8+ T cell count (/μL)	185	NA	693	206	536	NA
IL-6 (pg/mL)	NA	6.4	NA	3.6	48.1	NA
IL-8 (pg/mL)	NA	32	NA	41	22	NA
IL-10 (pg/mL)	NA	5.0	NA	5.0	5.0	NA
CSF^†^						
Opening pressure (mmH_2_0)	290	260	>330	155	290	NA
WBC count (/μL)	48	34	53	9	84	26
PMN count (/μL)	12	2	15	0	4	3
Protein (g/L)	0.63	0.62	NA	0.44	0.76	0.58
Glucose (mmol/L)	2.7	2.8	NA	3.2	3.3	2.9
IgG (mg/L)	49.2	138.0	NA	66.2	237.0	NA
IgG index	0.59	1.74	NA	1.01	2.16	NA
SOB	−	+	NA	+	+	NA
RF (IU/mL)	NA	NA	0	NA	NA	NA
IL-6 (pg/mL)	>1000	457.0	2.0	NA	244.0	NA
IL-8 (pg/mL)	288	294	42	NA	134	NA
IL-10 (pg/mL)	8.0	6.9	5.0	NA	7.7	NA
Important tests with negative results						
Peripheral blood	ANA, ANCA, APLA, ACE, IgG4, MOG, BAT	ANA, ANCA, APLA, ACE, IgG4, MOG, BAT, CrAg	ANA, ACE, IgG4, AQP4	ANA, ANCA, APLA, ACE, IgG4, AQP4	ANA, ANCA, APLA, ACE, IgG4, BAT, CrAg	ANA, ANCA, APLA, ACE, IgG4, BAT, CrAg
CSF	Cytology, Xpert, CrAg, bacterial culture, fungal culture, TB culture, Filmarray ME, mNGS	Cytology, Xpert, CrAg, bacterial culture, fungal culture, mNGS	Cytology, TB PCR, bacterial culture, fungal culture	Cytology, CrAg, bacterial culture	Cytology, Xpert, CrAg, bacterial culture, fungal culture, TB culture, mNGS	Cytology, TB PCR, Xpert, CrAg, bacterial culture, HSV PCR

ACE, angiotensin converting enzyme levels; ACPA, anticitrullinated peptide antibody; AKA, antikeratin antibody; ANA, antinuclear antibodies; ANCA, antineutrophil cytoplasmic antibodies; APF, antiperinuclear factor; APLA, antiphospholipid antibodies; AQP4, anti- aquaporin 4 antibody; BAT, Brucella agglutination test; CD, cluster of differentiation; CrAg, Cryptococcus antigen test; CSF, cerebrospinal fluid; ESR, erythrocyte sedimentation rate; hsCRP, high sensitivity C-reaction protein; HSV, herpes simplex virus; IgG, Immunoglobulin G; IgG4, immunoglobulin G4 levels; IL, interleukin; mNGS, metagenomic next-generation sequencing; MOG, anti-myelin oligodendrocyte glycoprotein antibody; NA, not available; PCR, polymerase chain reaction; PMN, polymorphonuclear neutrophil; RF, rheumatoid factor; SOB, specific oligoclonal bands; TB, tuberculosis; WBC, white blood cell. †, performed before the initiation of immunotherapy for RM.

Lumbar puncture was performed in all patients, and intracranial hypertension was detected in four. All patients showed mild to moderate pleocytosis in cerebrospinal fluid (CSF), and two patients had increased polymorphonuclear neutrophil counts. One patient tested negative for rheumatoid factor in CSF. IL-6 levels in CSF were tested in four patients and found to be dramatically elevated in three. IL-6 was higher in CSF than in serum in two tested patients (cases 2 and 5). Tests for other causes of meningitis yielded negative results (**Table 2**).

**Figure 2:**
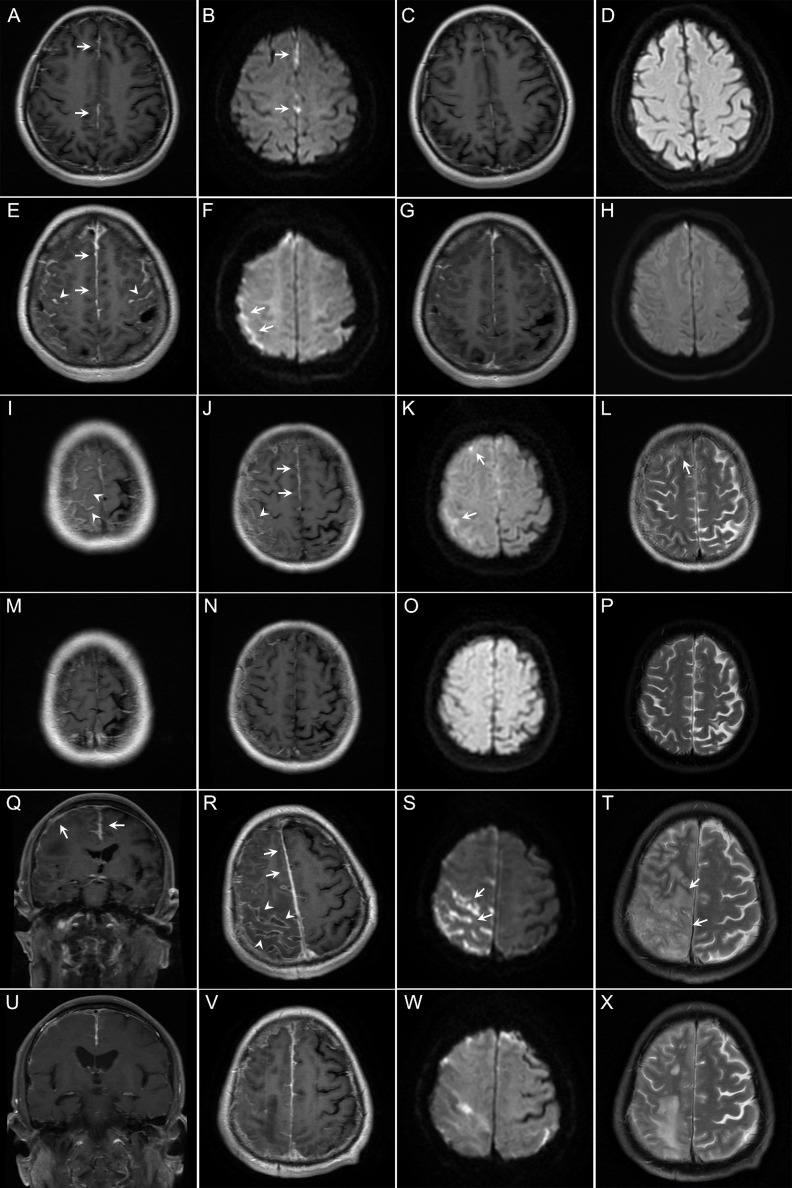
Findings on magnetic resonance imaging (MRI) of the brain in the patients with rheumatoid meningitis in this study. **(A–D)** In case 1, brain contrast MRI performed 4 months after onset of symptoms of meningitis showed pachymeningeal enhancement (**A**, arrows). Diffusion-weighted imaging (DWI) showed restricted diffusion (**B**, arrows). The patient experienced improvement, with resolution of the lesions 10 months after immunotherapy **(C, D)**. **(E–H)** In case 2, brain contrast MRI performed 5 months after onset of symptoms of meningitis showed enhancement of both the pachymeninges (**E**, arrows) and leptomeninges (**E**, arrowheads). Restricted diffusion was shown on DWI (**F**, arrows). Repeated MRI showed reduction of contrast enhancement and resolution of restricted diffusion 3 months after immunotherapy **(G, H)**. **(I–P)** In case 4, brain MRI performed 12 months after onset of symptoms of meningitis also showed involvement of both the pachymeninges (**J**, arrows) and leptomeninges (**I, J**, arrowheads) with restricted diffusion (**K**, arrows). T2-weighted MRI scans showed a small cortical lesion (**L**, arrow). Repeated MRI showed resolution of the lesions 28 months after immunotherapy **(M–P)**. **(Q–X)** In case 5, brain contrast MRI performed 3 months after onset of symptoms of meningitis revealed asymmetric involvement of both the pachymeninges (**Q, R**, arrows) and leptomeninges (**R**, arrowheads). Note the lesions are mainly located on the convex surface of the cerebral hemisphere. DWI showed sulcal restricted diffusion (**S**, arrows). T2-weighted MRI scans showed lesions in the parenchyma (**T**, arrows). Repeated MRI showed reduction of the lesions 3 months after immunotherapy **(U–X)**.

Magnetic resonance imaging (MRI) of the brain revealed that the meninges were affected in all patients, and that the cerebral parenchyma was affected in one patient (case 5). Enhancement of the pachymeninges (case 1, **Figures 2A–D**) or both the pachymeninges and leptomeninges (cases 2–6, **Figures 2E–H**) was shown in different patients. Lesions were generally located on the convex surface of the cerebral hemisphere with sparing of the meninges around the basal cisterns (cases 1–6). Dramatic asymmetric involvement of the meninges was observed in three patients (cases 3–5), all of whom had recurrent focal neurological deficits on the opposite side. Diffusion-weighted imaging (DWI) showed lesions with restricted diffusion along the adjacent subarachnoid space (cases 1–6, **Figure 2**). Hydrocephalus was shown in one patient (case 6). The meningeal and parenchymal lesions were significantly improved after immunotherapy, with gradual disappearance of hyperintensities on DWI. In case 5, repeated MRI of the brain showed a dynamic change. About one month after onset of meningeal symptoms, the meninges of the right parietal lobe were mainly affected with small (≤1cm) periventricular white matter lesions. About 2 months after onset, the meninges of the right frontal lobe were also affected with formation of confluent white matter lesions in the right parietal lobe. About 3 months after onset, most of the right parietal lobe was affected (**Figures 2Q–T**). After immunotherapy, the lesions were significantly reduced (**Figures 2U–X**).

### 3.3 Immunotherapy and outcomes

Details of the immunotherapy administered and outcomes are summarized in **Figure 1** and **Table 1**. RM occurred during DMT in four patients. Case 1 had a medical history of central serous chorioretinopathy and case 2 had a history of stage 3 hypertension, diabetes mellitus, and dyslipidemia and was overweight. Both these patients refused steroids. Case 5 was already receiving steroids for severe arthritis. Three patients (cases 1, 2, and 5) improved on tocilizumab induction therapy, and the other three (cases 3, 4, and 6) improved on pulse methylprednisolone induction therapy during the acute episode. The neurological symptoms in these patients improved dramatically within the first week. For maintenance therapy, one patient received tocilizumab and hydroxychloroquine, one received tocilizumab, mycophenolate mofetil and low-dose oral corticosteroids, one received adalimumab and methotrexate, and two received low-dose oral corticosteroids with an immunosuppressive agent.

One patient (case 5) had complex medical conditions. Before immunotherapy, his level of consciousness decreased, and he developed complete hemiplegia on the left side. He showed significant improvement after treatment with tocilizumab and steroids. He was able to walk and live independently about 2 months after immunotherapy. However, he experienced a traumatic hip fracture during exercise, after which he became bedridden despite an artificial femoral head replacement. His neurological symptoms worsened, and he received prolonged treatment with steroids (≥1mg/kg daily) for 2 months and monthly tocilizumab. After discharge, he showed some improvement of neurological symptoms. However, he finally died of *Pneumocystis jirovecii* pneumonia, a serious infective complication of immunosuppressive therapy. The other five patients had a good outcome, with an mRS score of 0–1 at the last follow-up. No relapse of RM occurred while the patients were on immunotherapy during a median follow-up of 16.5 months.

## 4 Discussion

This retrospective investigation has characterized the clinical and neuroimaging features of Chinese patients with RM and adds some new insights into this condition. First, patients with RM exhibited a range of clinical manifestations, including hyperacute focal neurological deficits and seizures, which served as crucial clues for the diagnosis. Second, Chinese patients with RM and the previously reported patients shared some striking neuroimaging features. Third, the patients responded well to appropriate immunotherapy. Tocilizumab might be effective as both induction and maintenance therapy in patients with RM.

RM is a rare neurological complication of RA. A retrospective study by Parsons et al. in 2020 identified 14 patients with RM within the previous 28 years at the Mayo Clinic (11). In 2021, Villa et al. conducted a systematic review in which they identified 130 patients with RM from 103 studies reported between 1954 and 2020 (14). In this study, we found that less than one percent of inpatients admitted with RA developed RM. This proportion of patients with RA who develop meningeal involvement might even be overestimated, given that some of our patients were specifically referred to our encephalitis center.

Diagnosis of RM is challenging. As in our patients, meningitis can occur as the initial clinical manifestation of RA or after decades of arthritis and can also occur in the absence of active arthritis. Furthermore, many of the patients developed meningitis when they are still on DMT, and the neuroimaging findings may resemble a subdural empyema (15), making CNS infection and drug-induced meningitis likely. Until now, no diagnostic criteria for RM have been established. Therefore, the diagnosis of RM relies on appropriate exclusion of infectious, neoplastic, and other autoimmune etiologies. Meningeal biopsy performed for the purposes of diagnosis and differential diagnosis might reveal three abnormal patterns: rheumatoid nodules, vasculitis, and nonspecific meningeal inflammation (16). A systematic review found that 72.5% of patients underwent biopsy, which showed rheumatoid nodules in 42.3% cases, nonspecific meningeal inflammation in 94.8%, and vasculitis in 16.5% (14). In a retrospective study of 10 patients who underwent biopsy, 90% showed nonspecific inflammation or granulomatous necrosis (11). Many patients lack the relatively specific pathological findings of rheumatoid nodules or vasculitis. Furthermore, meningeal biopsy is invasive. Therefore, RM requires other diagnostic clues for diagnosis.

Patients with RM have some distinctive clinical manifestations. The analysis of 130 patients with RM by Villa et al. revealed that the common clinical manifestations were focal neurological signs (64.6%), systemic symptoms (51.3%), episodic headache (50.4%), neuropsychiatric alterations (47.7%), seizure (40.2%), and joint manifestations (27.4%) (14). In our study, headache was the most common symptom in Chinese patients with RM. Focal neurological deficits, especially those of hyperacute onset (within several minutes), were striking symptoms and occurred in 67% of patients but were infrequent in patients with meningitis of other etiology. It is speculated that the underlying pathophysiology may involve cortical spreading depression induced by inflammation of the adjacent meninges (4). Meanwhile, epileptic seizures were also common symptoms and occurred in 50% patients. The presence of transient focal neurological deficits and epileptic seizures provide diagnostic clues for RM in patients with RA.

Patients with RM have some relatively specific neuroimaging features, which might be used as a diagnostic marker and help with differential diagnosis of other etiologies of meningitis. Our patients shared some remarkable neuroradiological manifestations (**Figure 2**), some have also been reported in other patients with RM (1, 15, 17, 18). First, RM can affect both the pachymeninges and leptomeninges, with the latter reported more frequently (60% vs 82.7%) (14). With disease progression, MRI might reveal involvement of the cerebral parenchyma. Repeated MRI in one patient showed dynamic changes from involvement of the meninges to involvement of the cerebral parenchyma. This propensity is different from that in some other rheumatology diseases, such as anti-neutrophil cytoplasm antibody-associated vasculitis and IgG4-related disease, in which involvement of the pachymeninges is predicted (19). Second, there is a hyperintensity signal (restricted diffusion) along the adjacent subarachnoid space on DWI (1, 15, 17, 18). In the previous studies, hyperintensity in the subarachnoid space on DWI was mainly observed in patients with bacterial or cryptococcal meningitis (20). However, all patients in our study had restricted diffusion on DWI. The CSF findings in patients with RM are markedly different from those in patients with bacterial or cryptococcal meningitis. Therefore, in patients with meningitis who show hyperintensity in the subarachnoid space on DWI, the CSF results will help to differentiate RM from bacterial meningitis and cryptococcal meningitis. Third, the lesions are located predominantly in the frontoparietal region, the convex surface of the brain, but spare the meninges around the basal cisterns (1, 15, 17, 18). A retrospective study by Parsons et al. showed that 12 (86%) of 14 patients had a frontoparietal predominance (11). Similarly, all patients in our study showed a frontoparietal predominance. This distribution is significantly different from that seen in tubercular meningitis, in which the meninges around the basal cisterns are usually affected. Fourth, involvement of the meninges can be unilateral or bilateral, when bilateral, there is usually lateral dominance. Unilateral involvement is more specific in RM. A retrospective study by Parsons et al. showed that asymmetric involvement was appreciated in 11 (78.6%) of 14 patients (11). Furthermore, three patients in this study showed asymmetric involvement. These neuroimaging features are helpful for the diagnosis of RM in patients with RA.

The evidence regarding treatment of RM is limited. It has been reported that CNS involvement occurs in patients with RA during immunotherapy (1, 9, 14, 21–25), such as corticosteroids (51%); non-biological DMTs—methotrexate, sulfasalazine, hydroxychloroquine, leflunomide, iguratimod, azathioprine, cyclosporine, bucillamine and tofacitinib (68%); biological DMTs—infliximab, etanercept and adalimumab (20%); and other agents (12%). However, RM has not been reported in patients on tocilizumab. In this study, RM occurred in four patients who showed active arthritis despite continuous corticosteroids and/or non-biological DMT for RA. IL-6 plays a role in induction and maintenance of the autoimmune process *via* B cell modulation and Th17 cell differentiation and in angiogenesis by upregulating the expression of intracellular adhesion molecules, which are important in the pathogenesis of RA (26). In patients with RA, high levels of IL-6/sIL-6R complex in synovial fluids are associated with joint destruction and disease progression (27). Tocilizumab is a monoclonal antibody that inhibits the IL-6 receptor, leading to inhibition of IL-6 signaling (28), and works rapidly and effectively in RA either as monotherapy or in combination with other agents (29–32). High IL-6 levels were also detected in the CSF of patients with RM in this study. Therefore, we speculated that tocilizumab might also be effective in the treatment of RM. In the previously reported cases, pulse corticosteroid therapy was the main induction therapy used for RM (14). Although application of tocilizumab in RM has rarely been reported, it has been used successfully alone or with methotrexate following corticosteroid therapy in four patients with RM (7, 22, 33). However, use of tocilizumab as induction therapy has not been reported. In our study, two patients received tocilizumab alone, and one (case 5) received tocilizumab with low-dose corticosteroids as induction therapy; all experienced rapid improvement, suggesting that tocilizumab could be an effective induction therapy for RM. Meanwhile, three patients received tocilizumab with another immunosuppressive agents as maintenance therapy; all of these patients responded well to treatment. Therefore, tocilizumab might be an effective induction and maintenance therapy for RM.

RM is a severe neurological complication of RA. Disease relapse has been reported in 31.2% of patients and had a lethal outcome in 14% (14). In our study, no relapse was observed but one patient died of an opportunistic infection. Patients treated with biological agents should be closely monitored for infectious diseases.

This retrospective study has some limitations. First, no patient underwent meningeal biopsy for diagnosis. Second, some important evaluations, such as DAS-28 and laboratory tests were not performed in all patients. Third, the DMT regimen was not consistent across all the patients.

## 5 Conclusion

Chinese patients with RM share some striking clinical and neuroimaging features, including hyperacute focal neurological deficits, predominant involvement of the meninges in the frontoparietal region, and hyperintensity signals along the adjacent subarachnoid space on DWI. Patients with RM respond well to appropriate immunotherapy. Tocilizumab could be a promising option for induction and maintenance therapy in RM.

## Data availability statement

The original contributions presented in the study are included in the article/supplementary material. Further inquiries can be directed to the corresponding author.

## Ethics statement

The studies involving human participants were reviewed and approved by institutional review board of Peking Union Medical College Hospital. Written informed consent for participation was not required for this study in accordance with the national legislation and the institutional requirements.

## Author contributions

SF and JZ, design of the study, drafting and revising of the manuscript, major role in the acquisition of data, and analysis of the data. BH, major role in the acquisition of data, analysis of the data, and revising of the manuscript. MGL, major role in the acquisition of data, revising of the manuscript. JN, YZ, CM, HR, and YCZ, major role in the acquisition of data and analysis of the data. FF, MTL, and XZ, analysis of the data, revising of the manuscript. HG, design of the study, drafting and revising of the manuscript, and analysis of the data. All authors contributed to the article and approved the submitted version.
